# Factors influencing collaboration between occupational health nurses and human resources personnel in supporting the return to work of employees with mental health problems: a cross-sectional study

**DOI:** 10.1093/joccuh/uiag039

**Published:** 2026-07-20

**Authors:** Naoko Murono, Rie Okamoto, Yukari Nakada, Satomi Ikeuchi, Yutaro Takahashi, Ryota Kumakura, Hiromi Yonezawa, Hisae Tsukada, Shizuko Omote

**Affiliations:** Doctoral Course of Graduate School of Medical Sciences, Kanazawa University, Kanazawa, Ishikawa, Japan; School of Nursing, Ishikawa Prefectural Nursing University, Ishikawa, Japan; Faculty of Health Sciences, Institute of Medical, Pharmaceutical and Health Sciences, Kanazawa University, Kanazawa, Ishikawa, Japan; Department of Nursing, Faculty of Health and Medical Sciences, Kyoto University of Advanced Science, Kyoto, Japan; Department of Nursing, Faculty of Medicine, Mie University, Mie, Japan; Faculty of Health Sciences, Institute of Medical, Pharmaceutical and Health Sciences, Kanazawa University, Kanazawa, Ishikawa, Japan; Faculty of Health Sciences, Institute of Medical, Pharmaceutical and Health Sciences, Kanazawa University, Kanazawa, Ishikawa, Japan; School of Nursing, Ishikawa Prefectural Nursing University, Ishikawa, Japan; School of Nursing, Ishikawa Prefectural Nursing University, Ishikawa, Japan; Faculty of Health Sciences, Institute of Medical, Pharmaceutical and Health Sciences, Kanazawa University, Kanazawa, Ishikawa, Japan

**Keywords:** return to work, mental health, interprofessional collaboration, occupational health nurse, human resources personnel, structural equation modeling

## Abstract

**Objectives:**

Collaboration between occupational health nurses and human resources (HR) personnel is essential in supporting the return to work of employees with mental health problems. However, the factors influencing this collaboration are unclear, and this study therefore aimed to examine these factors.

**Methods:**

A cross-sectional questionnaire survey was conducted among 3049 occupational health nurses from the Japan Society for Occupational Health, and responses from 528 participants were analyzed. Collaboration was measured using the Japanese version of the Relational Coordination Survey (J-RCS). Independent variables included individual factors relating to both occupational health nurses and HR personnel, relational factors, workplace climate factors, and organizational justice. Structural equation modeling was used to examine associations with J-RCS scores.

**Results:**

The mean J-RCS score was 3.58 (SD = 0.79), indicating a moderate level of collaboration. Structural equation modeling showed that relational factors (β = .75, *P* < .001) and organizational justice (β = .40, *P* = .003) had significant direct effects on collaboration. Individual factors relating to occupational health nurses or HR personnel, and workplace climate factors, showed no direct effects but contributed indirectly through associations with other factors. The model explained 88.8% of the variance in J-RCS scores.

**Conclusions:**

Enhancing the quality of relationships, including trust and information sharing, and improving organizational justice, are crucial for strengthening collaboration in support for return to work. This study provides practical intervention targets and useful insights for improving occupational health policies and practices.

## Teaser

**Key points**
 

- **What is already known on this topic:** Collaboration between occupational health nurses and human resources (HR) personnel is essential for effective return-to-work support for employees with mental health problems. However, little is known about the factors influencing this collaboration.
- **What this study adds:** This is the first nationwide study in Japan (*n* = 528) to comprehensively examine factors affecting collaboration using structural equation modeling. Relational factors and organizational justice had significant direct effects on collaboration. Workplace climate factors and individual factors relating to both occupational health nurses and HR personnel had no direct effects but contributed indirectly through their associations with other factors.
- **How this study might affect research, practice, or policy:** The findings indicate that building trust-based relationships and enhancing organizational justice are essential for strengthening collaboration between occupational health nurses and HR personnel in providing return-to-work support for employees with mental health problems. These insights may improve the quality of support available to these employees. They may also support interprofessional collaboration to improve return-to-work support for those with other health conditions and on broader occupational health activities.

## 1. Introduction

Depressive disorders affect more than 300 million people worldwide, and prevalence is increasing.^1^ Mental health–related absence tends to be prolonged and recurrent^2^^,^^3^ with widely varying severity and duration.^3^ It is also influenced by factors beyond the workplace.^1^^,^^3^^,^^4^ These issues reduce workplace productivity and labor participation.

Effective return-to-work (RTW) support systems are required to facilitate the reintegration of individuals not working because of illness or disability.^5^ World Health Organization (WHO) guidelines identify such support systems as central to mental health interventions.^3^ Effective support requires collaboration among stakeholders, including workers, occupational health nurses (OHNs), and human resources (HR) personnel.^3^^,^^6^ RTW support for mental health problems often requires individualized and gradual adjustments, including flexible working and job modifications, reflecting the fluctuating nature of mental health conditions.^4^^,^^7^^,^^8^ Collaboration between HR personnel and occupational health (OH) staff is therefore critical.^6^ However, their roles vary widely and frequently overlap,^4^ causing conflict and difficulties.^9^^,^^10^ A survey of HR personnel about RTW systems reported that approximately 80% of companies experienced difficulties managing employees with mental health problems.^11^

Japan’s Industrial Safety and Health Act mandates the appointment of occupational physicians in workplaces with ≥50 employees, but OHNs are not legally required.^12^ The number, roles, and institutional positioning of OHNs therefore vary. One survey found that about one-third of OHNs are the sole OHN in their workplace.^13^ However, another noted that when present, OHNs are generally accessible to workers and often the first point of contact for health concerns,^14^ typically collecting information from employers and employees, and coordinating among stakeholders.^14^ By contrast, HR personnel often lack medical expertise and rely on OH staff for specialized responses.^9^ Effective collaboration requires information sharing and trust. Friction arising from insufficient understanding may hinder relationship building.^10^

Previous studies on interprofessional collaboration in nursing identified 3 categories of influencing factors: individual, team, and organizational.^15^ Team factors often relate to the quality of relationships, and include trust, support, and respect among members. These insights may be applicable to collaboration between OHNs and HR personnel. At the individual level, a qualitative study in Europe reported that insufficient knowledge among supporters was a barrier in RTW programs for cancer survivors.^16^ Similarly, a better understanding of the role of OHNs among HR personnel can facilitate collaboration.^17^ At the relational level, discrepancies in supporters’ intentions can cause operational challenges.^18^^,^^19^ OHNs may hesitate to share information with HR personnel because of their ethical duty of individual confidentiality.^20^^,^^21^ Information-sharing is central to collaboration,^22^ and difficulties in sharing information about mental health interventions, reported by nearly 80% of OHNs, and by more than 60% in RTW support,^20^ may act as barriers. However, mutual trust and respect among professions promotes collaboration.^15^

At the organizational level, union members in Canada reported that a supportive workplace climate facilitated RTW after depression, whereas stigma delayed RTW.^23^ An integrative review also identified stigma as a barrier to RTW.^7^ Stigma and insufficient understanding of mental health problems may discourage employees from disclosing their condition or seeking support, complicating collaboration.^7^^,^^18^ Even with gradual support adjustments, RTW may fail without sufficient support.^7^ Workplace climate may therefore influence collaboration between OHNs and HR personnel. Organizational justice also affects turnover intention, organizational commitment, and job performance.^24^ HR personnel often occupy management positions and may even supervise OHNs,^17^ and organizational justice is therefore likely to affect collaboration.

Clarifying these factors may enhance RTW support and workplace health and productivity. Previous research has suggested that individual, relational, and organizational factors affect collaboration, but few studies have examined the effect of all these factors on collaboration between OHNs and HR personnel. This study therefore aimed to examine the associations between these factors and OHN–HR collaboration in RTW support for people with mental health problems. We hypothesized that (H1) OHN-related, (H2) HR-related individual factors, (H3) relational factors, (H4) workplace climate factors, and (H5) organizational justice would be positively associated with collaboration, and would also be positively intercorrelated. Mediated relationships were not formally hypothesized, but associations among these factors were considered during the analysis.

## 2. Methods

### 2.1 Study design and participants

This study used a cross-sectional questionnaire to survey OHNs (*n* = 3049) from the membership list of the Japan Society for Occupational Health (JSOH), with permission.

Exclusion criteria were clearly stated in the mailed survey invitation, and potential participants confirmed their eligibility before completing the questionnaire. The study excluded:

- Those not involved in RTW support for employees with mental health problems in their current workplace during the past year.
- Those engaged in OHN activities under employment contracts with multiple workplaces.

Some respondents discontinued the internet-based questionnaire before completion. Only completed questionnaires were included in the analysis.

This study did not restrict mental health problems to specific diagnoses or severity levels and did not limit RTW support to specific stages or activities, consistent with Japanese governmental guidelines.^6^^,^^25^ This definition of RTW support was also in line with the view that RTW is a continuous process from the beginning of leave through to post-return follow-up.^2^

### 2.2 Data collection

The invitation described the study objectives, methods, and the voluntary nature of participation. Participants were instructed to respond online via SurveyMonkey (SurveyMonkey Inc, San Mateo, CA, USA). Consent was obtained on the survey opening page, and responses were collected anonymously. The survey ran from March 12 to March 31, 2024.

### 2.3 Outcome measure (collaboration between OHNs and HR personnel)

Collaboration between OHNs and HR personnel was measured using the Japanese version of the Relational Coordination Survey (J-RCS).^26^ J-RCS has high reliability and validity, with Cronbach α ranging from .770 to .859.^26^ The underlying concept—relational coordination—is “a mutually reinforcing process of interaction between communication and relationships carried out for the purpose of task integration.”^27^ The RCS has been widely used to assess interprofessional collaboration across settings, including health care.^27–30^ It was therefore considered appropriate for OHNs working in diverse organizational contexts. RTW outcomes are strongly influenced by factors such as illness severity, organizational policies, job availability, and economic conditions, which are difficult to adequately control in a cross-sectional survey. However, collaboration between OHNs and HR personnel is a proximal and modifiable process directly relevant to RTW support provision, making it an appropriate outcome for examining factors influencing interprofessional practice.

The self-administered scale contains 7 items.^28^ Each item is rated on a 5-point Likert-type scale, and the mean of all 7 items is calculated. Scores <3.5 indicate weak relational coordination, 3.5-4.0 moderate, and >4.0 strong.^27^

### 2.4 Factors influencing collaboration

We used previous studies to develop items on individual, relational, and workplace climate factors that may influence collaboration between OHNs and HR personnel. All items were rated on a 4-point Likert-type scale, with some reverse-coded.

### 2.5 Individual factors

Individual factor items were created from previous studies.^16^^,^^17^ We assessed OHNs’ views about their own and HR personnel’s knowledge of RTW support for employees with mental health problems (Q1, Q4) and the experience of managing such cases (Q2, Q5). OHNs were also asked about their level of self-directed learning (Q3), and HR personnel’s understanding about general OHN activities (Q6) (1 = strongly disagree to 4 = strongly agree).

### 2.6 Relational factors

The relational factors in this study were designed to capture underlying barriers and facilitators to cooperation.^18–21^ Whereas the J-RCS assessed the quality of collaboration itself in RTW support for workers with mental health problems, the relational factors assessed OHNs’ perceptions of everyday conditions that may facilitate or hinder such collaboration. Participants were asked about discrepancies in policies or opinions between OHNs and HR personnel (Q7), and difficulties in sharing health information (Q8) (1 = often to 4 = never). Q8 assessed perceived difficulty in information sharing, whereas J-RCS items 1-3 assessed communication frequency, timeliness, and accuracy within collaborative practice. To assess HR support, 2 items adapted from the New Brief Job Stress Questionnaire (short version, 80 items)^31^ were used: “How freely can you talk with HR personnel?” (Q9) and “How reliable are HR personnel when you are troubled?” (Q10) (1 = not at all to 4 = extremely). Q9 and Q10 assessed the perceived accessibility and reliability of HR personnel as support sources, whereas J-RCS items 4 and 7 assessed problem-solving and mutual respect within collaborative practice.

### 2.7 Workplace climate factors

Items on workplace climate factors were drawn from previous studies.^7^^,^^17^^,^^23^^,^^24^ Participants were asked whether it is more challenging to provide RTW support for employees with mental health problems than other illnesses (Q11; 1 = often to 4 = never), and about their workplace’s acceptance of mental health problems (Q12; 1 = negative to 4 = positive), willingness to cooperate in RTW support (Q13; 1 = uncooperative to 4 = cooperative), and whether RTW support systems were established (Q14; 1 = not established at all to 4 = fully established).

### 2.8 Organizational justice

Organizational justice was assessed using the Japanese version of the Organizational Justice Questionnaire.^32^^,^^33^ This scale has high reliability (Cronbach α = .85-.94) and contains 7 items on procedural justice (PJ) and 6 items on interactional justice (IJ). Each item was rated on a 5-point Likert-type scale, and mean scores were calculated.

### 2.9 Other characteristics

Demographic variables included gender (male, female, or prefer not to answer), age (5 categories: 20-29, 30-39, 40-49, 50-59, and 60+ years old), qualifications, employment status (full-time or part-time), contract type (permanent or nonpermanent), years of OHN experience, and years working in the current organization. Work-related characteristics included type of institution, the workplace’s main industry, and number of employees. Industry classification used the Japan Standard Industrial Classification.^34^

### 2.10 Statistical analysis

Overall, 528 participants were included. The structural equation model included 6 latent variables (J-RCS and 5 predictors) and was overidentified. Sample size adequacy was evaluated by model complexity. An a priori power analysis using *semPower 2*,^35^ based on root mean square error of approximation (RMSEA) = 0.05, α = .05, power = 0.90, *df* = 170, and 21 manifest variables, indicated a required sample size of 147; the actual sample size was 528.

Descriptive statistics were calculated for participant characteristics and variables, and missing values and normality were assessed. Skewness and kurtosis indicated no substantial deviations from normality. For the 14 study-developed items, exploratory factor analysis (EFA) was conducted using the principal factor method with promax rotation, because factor correlations were expected. Score distributions were examined for ceiling and floor effects. Sampling adequacy was evaluated using the Kaiser-Meyer-Olkin (KMO) index and Bartlett test of sphericity. Factor extraction was based on eigenvalues ≥1.0, scree plots, and factor loadings ≥0.40. Reverse-coded items (Q7, Q8, Q11) were recoded before analysis, and internal consistency was assessed using Cronbach α. EFA was limited to the original items developed in this study; PJ and IJ were excluded because they are validated subscales with established factor structures.

The EFA-derived factor structure, together with the J-RCS and Organizational Justice Questionnaire (PJ and IJ), was tested using confirmatory factor analysis (CFA) with maximum likelihood estimation. Model fit was evaluated using RMSEA <0.08, standardized root mean square residual (SRMR) <0.08, comparative fit index (CFI) ≥0.90, Tucker-Lewis index (TLI) ≥0.90, and goodness-of-fit index (GFI) ≥0.90.

A theory-informed hypothetical model, developed from previous theoretical and empirical literature, posited associations between individual factors for OHNs and HR personnel, relational factors, workplace climate factors, organizational justice, and OHN–HR collaboration. Structural equation modeling (SEM) examined these associations, with J-RCS as the dependent variable, the 5 factors as independent variables, and covariances specified among all independent variables. Additional analyses assessed discriminant validity, common method bias, and model robustness (Supplementary Data 1-3). SEM fit was evaluated using the same indices as the CFA. All analyses used IBM SPSS Statistics version 29.0 and IBM SPSS *Amos* version 29.0 (IBM Corp, Armonk, NY, USA), with a significance level of 5% (2-tailed).

### 2.11 Ethical considerations

Potential participants were mailed once using address labels provided by the JSOH. The remaining labels were shredded after mailing in accordance with Society regulations. The survey was anonymous and conducted with informed consent; no personally identifiable information, including IP addresses, was collected. All procedures complied with the Declaration of Helsinki. This study was approved by the Ethics Committee of Ishikawa Prefectural Nursing University (No. 2023-447-2) and the Medical Ethics Review Committee of Kanazawa University (No. 2023-209).

## 3. Results

### 3.1 Descriptive statistics and reliability of measures

**Figure 1:**
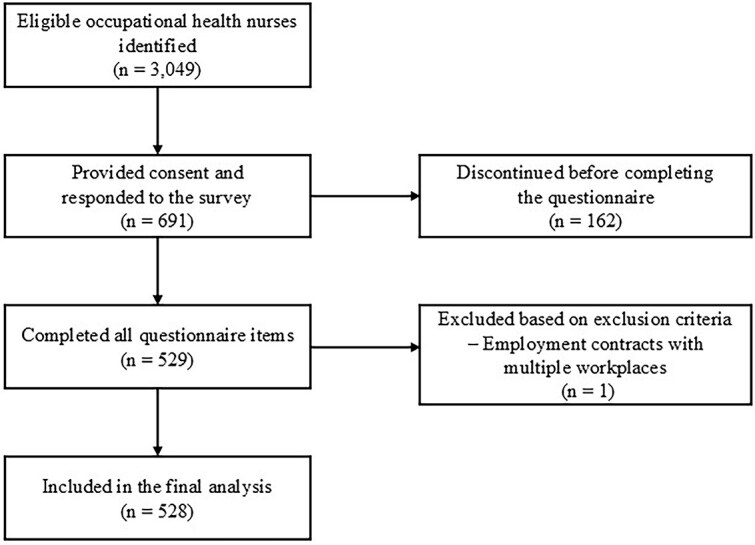
Flow diagram of participant selection. Note. The online questionnaire required responses to all items before submission; therefore, item-level missing data did not occur among completed questionnaires.

Of the 3049 eligible participants, 691 provided consent and started the survey, and 529 completed all questionnaire items. One respondent meeting the exclusion criteria was subsequently excluded, leaving data from 528 participants (response rate: 17.3%) for analysis (Figure 1). Table 1 shows their characteristics. Cronbach α coefficient was .903 for the J-RCS, .913 for PJ and .943 for IJ, all demonstrating excellent reliability. The mean J-RCS score was 3.58 (SD = 0.79), showing a moderate level of collaboration (Table 2).

**Table 1:** Demographic and organizational characteristics of the respondents.

	*n*	%
**Gender**		
**Male**	10	1.9
**Female**	514	97.3
**Prefer not to answer**	4	0.8
**Age group, y**		
**20-29**	23	4.4
**30-39**	109	20.6
**40-49**	190	36.0
**50-59**	160	30.3
**60+**	46	8.7
**Licensed Public Health Nurse (Japan)**		
**Yes**	448	84.8
**No**	80	15.2
**Employment status**		
**Full-time**	485	91.9
**Part-time**	43	8.1
**Contract type**		
**Permanent employee**	374	70.8
**Contract employee**	111	21.0
**Temporary agency worker**	9	1.8
**Part-time worker**	17	3.2
**Other**	17	3.2
**Years of experience as an OHN (mean ± SD)**	15.3 ± 9.7	
**Years of service in the current organization (mean ± SD)**	11.2 ± 9.3	
**Type of affiliation**		
**Enterprise**	453	85.8
**Health insurance society**	32	6.1
**Other**	43	8.1
**Industry type** ^**a**^		
**Construction**	21	4.0
**Manufacturing**	235	44.5
**Electricity, gas, heat supply, and water**	23	4.4
**Information and communications**	60	11.4
**Transportation and postal services**	25	4.7
**Wholesale and retail trade**	33	6.3
**Finance and insurance**	20	3.8
**Medical, health care and welfare**	30	5.7
**Services, not elsewhere classified**	20	3.8
**Others**	61	11.4
**Number of employees**		
**<50**	23	4.3
**50-499**	134	25.4
**500-999**	88	16.7
**1000-2999**	142	26.9
**3000+**	141	26.7

Abbreviation: OHN, occupational health nurse.

^a^Industry categories were classified using the Japanese Standard Industrial Classification (20 divisions). Categories with fewer than 20 respondents were grouped together as “Others.”

**Table 2:** Descriptive statistics, internal consistency, and composite scores of the J-RCS, Organizational Justice Scale, and main independent variables.

Items		Mean	SD	Cronbach α
**J-RCS score (overall)**		3.58	0.79	.903
**RC1**	**Frequent communication**	3.96	1.38	–
**RC2**	**Timely communication**	3.29	0.94	–
**RC3**	**Accurate communication**	3.44	0.93	–
**RC4**	**Problem-solving communication**	4.02	0.82	–
**RC5**	**Shared goals**	3.26	1.03	–
**RC6**	**Shared knowledge**	3.45	0.80	–
**RC7**	**Mutual respect**	3.66	0.96	–
**Organizational justice**				
** PJ**	**Procedural justice**	3.63	0.77	.913
** IJ**	**Interactional justice**	3.74	0.88	.943
**Composite scores of latent variables**		**Mean**	**SD**	**Min-Max**
**Individual factors relating to OHNs (range: 1-4)**		3.12	0.50	1.33-4.00
**Individual factors relating to HR personnel (range: 1-4)**		2.44	0.67	1.00-4.00
**Relational factors (range: 1-4)**		2.61	0.56	1.00-4.00
**Workplace climate factors (range: 1-4)**		2.91	0.57	1.00-4.00
**Organizational justice (range: 1-5)**		3.68	0.73	1.07-5.00

Abbreviations: HR, human resources; J-RCS, Japanese version of the Relational Coordination Survey; OHN, occupational health nurse; RC, Relational Coordination item.

Composite scores represent factor-based mean scores used in the structural model.

### 3.2 Exploratory factor analysis

Q14 was excluded from the EFA because it showed a ceiling effect. No items met the criteria for floor effects. The KMO measure for the EFA on the remaining 13 items was 0.777, and Bartlett test of sphericity was significant (*P* < .001), confirming sampling adequacy. Factors with eigenvalues ≥1 were extracted using the principal factor method with promax rotation. Q11, which had a factor loading <0.40, was excluded. A 4-factor, 12-item structure was identified, explaining 57.6% of the total variance. Cronbach α coefficients for the 4 factors were .782, .725, .881, and .757. Inter-factor correlations ranged from 0.10 to 0.62 (Table 3). Factor 1 was interpreted as relational factors, Factor 2 as individual factors for OHNs, Factor 3 as individual factors for HR personnel, and Factor 4 as workplace climate factors. Composite scores for each latent variable were calculated (Table 2).

**Table 3:** Factor loadings of the 14 items from the exploratory factor analysis (promax rotation, *n* = 528).

No.	Item	Factor 1	Factor 2	Factor 3	Factor 4
**Q9**	How freely can you talk with HR personnel?	0.928	–	–	–
**Q10**	How reliable are HR personnel when you are troubled?	0.772	–	–	–
**Q6**	To what extent do HR personnel understand occupational health nursing activities in general?	0.446	–	–	–
**Q7**	To what extent do differences in policies or opinions arise between you and HR personnel regarding return-to-work support for employees with mental health problems?	0.414	–	–	–
**Q8**	To what extent have you experienced difficulties, hesitation, or dilemmas regarding whether and how to share personal health information of employees with mental health problems with HR personnel?	0.403	–	–	–
**Q2**	To what extent do you have knowledge about supporting employees with mental health problems?	–	0.922	–	–
**Q1**	To what extent do you have knowledge about mental health problems and their treatments?	–	0.839	–	–
**Q3**	To what extent do you engage in professional development on supporting employees with mental health problems (eg, at academic conferences or study meetings)?	–	0.412	–	–
**Q5**	To what extent do HR personnel have knowledge about supporting employees with mental health problems?	–	–	0.947	–
**Q4**	To what extent do HR personnel have knowledge about mental health problems and their treatments?	–	–	0.828	–
**Q13**	To what extent do you think your workplace is cooperative toward the return-to-work of employees with mental health problems?	–	–	–	0.849
**Q12**	How does your organization perceive employees with mental health problems?	–	–	–	0.719
	Eigenvalue	4.220	1.938	1.174	1.024
	Variance explained, %	32.20	13.46	6.59	5.36
	Cumulative variance explained, %	32.20	45.66	52.25	57.61
	Cronbach α	.782	.725	.881	.757
**No.**	**Items excluded from the factor solution**	**Reason for exclusion**			
**Q11**	To what extent is return-to-work support for employees with mental health problems more challenging compared with that for employees with other illnesses?	Low factor loading (0.397 < 0.40)			
**Q14**	To what extent is your workplace organized with formalized support systems for return-to-work of employees with mental health problems (eg, written procedures)?	Ceiling effect present			

### 3.3 Confirmatory factor analysis

CFA was used to examine the validity of the measurement model with the 4 factors from the EFA, J-RCS, PJ, and IJ. Based on modification indices ≥20, error covariances were added only between items within the same latent variable when theoretically justified. The final model demonstrated good fit: RMSEA = 0.050, SRMR = 0.040, CFI = 0.963, TLI = 0.954, and GFI = 0.930. Table 4 shows correlations among latent variables. Additional assessments of discriminant validity and common method bias are presented in Supplementary Data 1 and 2, respectively.

**Table 4:** Pearson correlations among the latent variables.

		1	2	3	4	5	6
**1**	**J-RCS**	–	–	–	–	–	–
**2**	**Individual factors for OHNs**	0.221^***^	–	–	–	–	–
**3**	**Individual factors for HR personnel**	0.633^***^	0.221^***^	–	–	–	–
**4**	**Relational factors**	0.919^***^	0.222^***^	0.752^***^	–	–	–
**5**	**Workplace climate factors**	0.522^***^	0.099	0.436^***^	0.542^***^	–	–
**6**	**Organizational justice**	0.844^***^	0.169^**^	0.589^***^	0.811^***^	0.741^***^	–

Abbreviations: J-RCS, Japanese version of the Relational Coordination Survey; OHN, occupational health nurse. ^**^ *P* < .01, ^***^P < .001.

### 3.4 Structural equation modeling

Overall fit indices for the SEM were equivalent to those of the CFA: RMSEA = 0.050, SRMR = 0.040, CFI = 0.963, TLI = 0.954, and GFI = 0.930. Relational factors (β = .75, *P* < .001) and organizational justice (β = .40, *P* = .003) showed significant direct effects on collaboration. Individual factors for OHNs (β = .03, *P* = .416) and HR personnel (β = −.11, *P* = .090), and workplace climate factors (β = −.13, *P* = .070) had no significant direct effects, but their correlations with other factors suggested potential indirect contributions.

**Figure 2:**
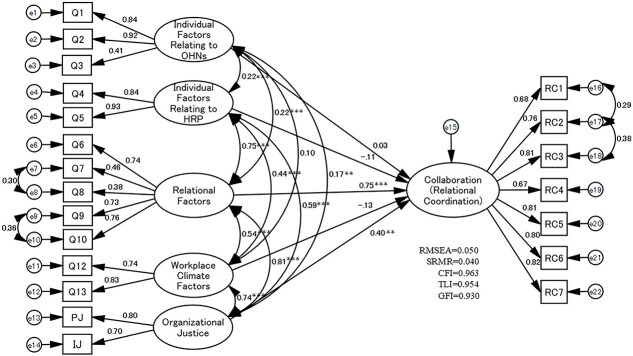
Structural equation model of factors influencing collaboration between occupational health nurses and human resources personnel, with standardized path coefficients. ^**^P < .01, ^***^P < .001. HRP, human resources personnel; IJ, interactional justice; OHN, occupational health nurse; PJ, procedural justice.

There were significant positive associations for all pairs of latent variables, except individual factors relating to OHNs and workplace climate factors. The strongest correlations were between relational factors and organizational justice (*r* = 0.81), relational factors and individual factors for HR personnel (*r* = 0.75), and workplace climate factors and organizational justice (*r* = 0.74) (all *P* < .001). There were moderate positive correlations between individual factors for HR personnel and both organizational justice (*r* = 0.59) and workplace climate factors (*r* = 0.44), and between relational factors and workplace climate factors (*r* = 0.54) (all *P* < .001) (Figure 2). The additional SEM with control variables showed acceptable model fit, and the main findings remained essentially unchanged, with similar standardized path coefficients for the main constructs (changes within approximately ±0.01; Figure S1).

The model explained 88.8% of the variance in J-RCS scores (*R*^2^ = 0.888), with relational factors and organizational justice making the largest contributions. The results therefore confirmed hypotheses H3 and H5, but not H1, H2, or H4.

## 4. Discussion

This study provided quantitative evidence about factors influencing collaboration between OHNs and HR personnel in supporting the RTW of employees with mental health problems, using data from a nationwide cross-sectional survey of OHNs in Japan. To our knowledge, this is the first study to do this. Relational factors and organizational justice had significant direct effects on collaboration. Individual factors for OHNs or HR personnel and workplace climate factors had no direct effects, but appeared to contribute indirectly through associations with other factors. The model explained 88.8% of the variance in J-RCS scores, indicating that these factors collectively accounted for most of the variability in collaboration.

Relational factors were particularly important for collaboration (β = .75). These include information-sharing, trust, and mutual understanding. A previous study suggested that proactive communication, trust, and mutual understanding positively influence collaboration.^36^ Some relational factors are conceptually close to elements of relational coordination, particularly those related to trust and communication. In this study, relational factors were theoretically positioned as conditions that may facilitate or hinder collaboration, whereas the J-RCS was used to measure the quality of collaboration itself. This distinction is consistent with relational coordination theory, which conceptualizes relational conditions such as trust and psychological safety as foundational elements supporting effective coordination.^27^ There was also a strong positive correlation between relational factors and organizational justice (*r* = 0.81). This aligns with the results of previous studies that suggested that trust and cooperation between OHNs and HR personnel are more likely to be present in workplaces with fair decision-making and equitable treatment.^37^ Overall, these findings extend organizational justice theory by demonstrating that fair organizational processes foster the relational conditions underpinning effective interprofessional collaboration. They extend previous qualitative research emphasizing trust, mutual understanding, role clarity, and fairness in RTW support by providing quantitative evidence of structural relationships between factors.^10^^,^^16^ Practically, strengthening organizational justice—for example, by clarifying decision-making procedures in RTW support—may enhance collaboration by fostering trust and relational quality. This may be particularly important for managing mental health problems, where individual variability and stigma can influence decision-making. However, challenges in balancing standardized procedures with case-specific flexibility suggest the need for further empirical examination.

These results suggest that strategic interventions targeting both relational factors and organizational justice can strengthen collaboration in RTW support. The strong positive correlation between these factors suggests that enhancing organizational justice may also improve relational factors. Developing fair decision-making processes and evaluation systems could therefore foster stronger relationships and enhance collaboration.

A strong positive correlation (*r* = 0.75) was also found between individual HR-related factors and relational factors, suggesting that the knowledge and attitudes of HR staff about mental health problems are linked to the quality of relationships with OHNs. However, the correlation between individual OHN-related factors and relational factors was relatively low (*r* = 0.22). Expertise and knowledge alone may therefore be insufficient to improve relational quality. Instead, HR personnel’s willingness to cooperate, and their understanding of OHN roles, may have more influence. This highlights the importance of enhancing both OHNs’ skills and HR personnel’s knowledge to improve collaboration. Randomized controlled trials of mental health training for managers found improved supportive behaviors,^38^ consistent with our findings that education for HR personnel is essential. WHO guidelines on workplace mental health also recommend managerial training to foster supportive behaviors.^3^ It seems likely that systematic training for HR personnel would improve support, especially because interprofessional education on mental health can reduce stigma.^39^

Organizational justice and workplace climate factors were also strongly correlated (*r* = 0.74), suggesting that workplace attitudes toward mental health problems and fairness in decision-making or interpersonal treatment are closely related.^33^ A meta-analysis showed that fair treatment is a key determinant of perceived organizational support.^40^ Although workplace climate factors had no direct effect on collaboration, they may function as contextual conditions that enable effective interpersonal relationships, with their influence potentially operating through relational factors.

Individual factors for HR personnel showed a nonsignificant weak negative coefficient (β = –.11) with collaboration. Previous studies suggested that overlapping roles and responsibilities between professional groups may threaten professional autonomy, generate tension, and hinder collaboration.^9^^,^^10^ In Japanese organizations, OHNs may sometimes be positioned within HR departments, which can blur professional roles. Under these circumstances, greater mental health–related knowledge among HR personnel may not necessarily strengthen collaboration, and may instead reflect or intensify role overlap with OHNs. Relational factors were included in the model, so this result shows a relative effect after controlling for relationship quality. Where relational foundations are insufficient, greater individual knowledge may become more important and be associated with weaker collaboration. This finding suggests that professional knowledge does not automatically lead to collaboration, but is contingent on the quality of relationships, supporting a relational perspective on interprofessional collaboration.

The model explained 88.8% of the variance in J-RCS scores, indicating high explanatory power. This finding should be interpreted cautiously, as the high *R*^2^ and the strong path from relational factors to the J-RCS (β = .75) may partly reflect item-level similarities between the constructs, particularly their shared focus on communication and trust. Nevertheless, the constructs were operationally distinct, and supplementary analyses provided some support for acceptable discriminant validity and did not suggest substantial common method bias. Relational factors captured OHNs’ perceptions of everyday conditions that may facilitate or hinder collaboration, whereas the J-RCS assessed the quality of collaboration itself in RTW support. Conceptual proximity and the cross-sectional, self-reported design should be considered when interpreting the strength of the observed associations.

This study has several limitations. First, its cross-sectional design precludes direct inference of causality. Relational factors were theoretically positioned as conditions that may facilitate or hinder collaboration, but their temporal precedence relative to the J-RCS could not be empirically established. Reciprocal or reverse causal relationships, especially between relational factors and collaboration, cannot be ruled out. Item-level similarities between the relational factors and the J-RCS may also have contributed to the strong path coefficient and high explanatory power observed. Possible overfitting and endogeneity should therefore be considered when interpreting the findings. Second, the study involved only OHNs in Japan, limiting generalizability to different cultural or organizational contexts. All participants were members of the JSOH, and thus may have been more professionally engaged or work in better-resourced organizations. The level of collaboration may therefore not be fully representative of OHNs in Japan. The findings are most applicable to organizations where OHNs and HR personnel have differentiated roles and work together on an ongoing basis, and less applicable where OH and HR functions are integrated into a single role, or collaboration is temporary with limited opportunities to build relationships. Third, mental health issues were not classified by severity or diagnostic category. The findings should therefore be interpreted as reflecting general tendencies, and not as practices specific to particular diagnoses or levels of severity. Fourth, the response rate was relatively low (17.3%), raising the possibility of response bias due to self-selection. Participation may have been greater from either OHNs with greater interest in or experience of collaboration or those perceiving greater challenges in collaboration, with opposite effects. Future studies should address these issues.

Future research should validate the model in other settings and investigate additional factors influencing collaboration. Longitudinal designs, including cross-lagged analyses, and intervention studies, could examine causal direction and potential reciprocal relationships between relational factors and collaboration. Mixed-method designs may provide deeper understanding by integrating quantitative and qualitative perspectives. Multi-source studies including HR personnel would provide a more comprehensive understanding. Finally, factors such as relational quality and organizational justice may be relevant to RTW support across chronic conditions, whereas workplace acceptance of mental health problems may be mental health-specific. Both types should be examined further.

## 5. Conclusions

To our knowledge, this is the first study to comprehensively examine factors influencing collaboration between OHNs and HR personnel in supporting the RTW of employees with mental health problems, using nationwide data and SEM. Relational factors and organizational justice had significant direct effects on collaboration. Workplace climate factors and individual factors for OHNs and HR personnel contributed indirectly through associations with other factors.

These findings suggest that building trust-based relationships and enhancing organizational justice will improve the quality of RTW support for employees with mental health problems. This study has practical implications and provides novel insights to inform future occupational health policies and practices.

## Supplementary Material

Supplementary_materials_uiag039

## Data Availability

The data that support the findings of this study are not publicly available because they contain information that could compromise the privacy of research participants. However, anonymized data are available from the corresponding author upon reasonable request.
