# Supplementary material for: Factors influencing collaboration between occupational health nurses and human resources personnel in supporting the return to work of employees with mental health problems: a cross-sectional study
Source: J Occup Health. 2026 Jul 20;68(1):uiag039. doi: 10.1093/joccuh/uiag039 (PMC13464725; doi:10.1093/joccuh/uiag039)
Supplement: Supplementary file 1 [file supplementary_materials_uiag039.zip › 260710 JOH-2025-0329-OA_Supplementary Data3_clean file.docx]

**Supplementary Data 3. Robustness analysis with control variables**

**Methods**

Given the cross-sectional design of the study, an additional structural equation model (SEM) including control variables was estimated to assess the robustness of the hypothesized associations. Eight background variables were included as covariates: gender, age, possession of a public health nurse qualification, employment status, contract type, years of experience, type of affiliation, and company size (number of employees). Years of service was excluded because of its high correlation with years of experience (Pearson’s r = 0.70). Industry type was also excluded because of small cell sizes that could result in unstable parameter estimates.

**Coding of control variables**

Gender was dichotomized for analysis. Age was treated as an ordinal categorical variable ranging from 1 (20–29 years) to 5 (60 years or older) and was entered as a single observed variable in the model. Years of experience was treated as a continuous variable.

Company size (number of employees) was categorized as fewer than 1,000 employees versus 1,000 or more employees. This categorization reflects Japanese occupational health regulation requirings the appointment of a full-time occupational physician in enterprises with 1,000 or more employees.^12^

Contract type was dichotomized as permanent versus non-permanent employee. This recategorization was applied because several original categories (contract employee, temporary agency worker, part-time worker, and other) had small cell sizes, which could lead to unstable parameter estimates in the SEM.

Type of affiliation was dichotomized as enterprise versus non-enterprise. This recategorization was applied because the number of participants affiliated with health insurance societies or other organizations was relatively small, and combining these categories improved analytical stability.

Employment status (full-time vs. part-time) and possession of a public health nurse qualification were included as categorical variables.

**Supplementary Figure S1**. Structural equation model adjusted for eight control variables.

** p < 0.01, p < 0.001

Note. OHN: occupational health nurse, HRP: human resources personnel, PHN: public health nurse, PJ: procedural justice, IJ: interactional justice

Paths from control variables and covariances among control variables are not displayed for clarity, although they were included in the model estimation.
