# Supplementary material for: Factors influencing collaboration between occupational health nurses and human resources personnel in supporting the return to work of employees with mental health problems: a cross-sectional study
Source: J Occup Health. 2026 Jul 20;68(1):uiag039. doi: 10.1093/joccuh/uiag039 (PMC13464725; doi:10.1093/joccuh/uiag039)
Supplement: Supplementary file 1 [file supplementary_materials_uiag039.zip › 260710 JOH-2025-0329-OA_Supplementary Data1_clean file.docx]

**Supplementary Data 1. Discriminant validity assessment**

**Method**

To examine discriminant validity, we used both the Fornell–Larcker criterion^S1^ and the heterotrait–monotrait ratio of correlations (HTMT) ^S2^. For the Fornell–Larcker criterion, the square root of the average variance extracted (AVE) for each construct was compared with the inter-construct correlations and values greater than the corresponding inter-construct correlations were considered to support discriminant validity.^S1^ Recent methodological literature has indicated that the Fornell–Larcker criterion may have limited sensitivity in detecting a lack of discriminant validity in some contexts; therefore, HTMT was used as a more stringent assessment. ^S2^ HTMT values below 0.90 were considered to indicate acceptable discriminant validity for conceptually similar constructs. ^S2^

**Results**
The Fornell–Larcker criterion was not fully met because the square roots of the AVE were lower than certain inter-construct correlations, particularly among J-RCS, relational factors, and organizational justice (Table S1). However, the HTMT criterion supported discriminant validity. All HTMT values were below the recommended threshold of 0.90, with the highest value observed between J-RCS and relational factors (HTMT = 0.877; Table S2). These findings suggest that, despite some conceptual proximity among the constructs, the HTMT results supported acceptable discriminant validity.

**Table S1**. Fornell–Larcker criterion for discriminant validity

| Construct | | 1 | 2 | 3 | 4 | 5 | 6 |
| --- | --- | --- | --- | --- | --- | --- | --- |
| 1 | J-RCS | **0.765** |  |  |  |  |  |
| 2 | Individual factors for OHNs | 0.221 | **0.759** |  |  |  |  |
| 3 | Individual factors for HR personnel | 0.633 | 0.221 | **0.890** |  |  |  |
| 4 | Relational factors | 0.919 | 0.222 | 0.752 | **0.635** |  |  |
| 5 | Workplace climate factors | 0.522 | 0.099 | 0.436 | 0.542 | **0.786** |  |
| 6 | Organizational justice | 0.844 | 0.169 | 0.589 | 0.811 | 0.741 | **0.750** |

Note. Diagonal values in bold indicate the square root of the average variance extracted (AVE). Off-diagonal values indicate inter-construct correlations.

**Table S2**. Heterotrait–monotrait ratio of correlations among latent constructs

| Construct | | 1 | 2 | 3 | 4 | 5 | 6 |
| --- | --- | --- | --- | --- | --- | --- | --- |
| 1 | J-RCS | **-** |  |  |  |  |  |
| 2 | Individual factors for OHNs | 0.246 | **-** |  |  |  |  |
| 3 | Individual factors for HR personnel | 0.623 | 0.240 | **-** |  |  |  |
| 4 | Relational factors | 0.877 | 0.228 | 0.702 | **-** |  |  |
| 5 | Workplace climate factors | 0.513 | 0.083 | 0.446 | 0.521 | **-** |  |
| 6 | Organizational justice | 0.830 | 0.177 | 0.589 | 0.802 | 0.755 | **-** |

**Supplementary references**

S1. Fornell C, Larcker DF. Evaluating structural equation models with unobservable variables and measurement error. *J Mark Res*. 1981;18(1):39-50. https://doi.org/10.2307/3151312

S2. Henseler J, Ringle CM, Sarstedt M. A new criterion for assessing discriminant validity in variance-based structural equation modeling. *J Acad Mark Sci*. 2015;43(1):115-135. https://doi.org/10.1007/s11747-014-0403-8
