# Supplementary material for: Factors influencing collaboration between occupational health nurses and human resources personnel in supporting the return to work of employees with mental health problems: a cross-sectional study
Source: J Occup Health. 2026 Jul 20;68(1):uiag039. doi: 10.1093/joccuh/uiag039 (PMC13464725; doi:10.1093/joccuh/uiag039)
Supplement: Supplementary file 1 [file supplementary_materials_uiag039.zip › 260710 JOH-2025-0329-OA_Supplementary Data2_clean file.docx]

**Supplementary Data 2. Assessment of common method bias**

**Method**

The potential impact of common method bias was assessed using Harman’s single-factor test, with a common latent factor approach as a supplementary assessment.^S3^ In Harman’s single-factor test, all observed items included in the measurement model were entered into an unrotated exploratory factor analysis. Common method bias was considered a potential concern if a single factor emerged or if the first unrotated factor explained a large portion of the total variance. In the supplementary common latent factor approach, a common latent factor was added to the confirmatory factor analysis model, with paths specified from the common latent factor to all observed items.^S3^ Standardized factor loadings from models with and without the common latent factor were compared to assess the extent to which the inclusion of the common latent factor altered the measurement parameters.

**Results**
In Harman’s single-factor test using principal factor analysis without rotation, five factors with eigenvalues greater than 1.0 were extracted. The first unrotated factor accounted for 41.1% of the total variance, indicating that no single factor accounted for a large portion of the variance. In the supplementary common latent factor approach, the absolute differences in standardized factor loadings between the models with and without the common latent factor ranged from 0.000 to 0.006, indicating minimal changes in the measurement parameters. These findings suggested a low likelihood of substantial common method bias.

**Supplementary references**

S3. Podsakoff PM, MacKenzie SB, Lee JY, Podsakoff NP. Common method biases in behavioral research: a critical review of the literature and recommended remedies. *J Appl Psychol*. 2003;88(5):879-903. https://doi.org/10.1037/0021-9010.88.5.879
